# Hypotension and Renal Dysfunction: The Ghosts of Heart Failure

**DOI:** 10.5935/abc.20170110

**Published:** 2017-08

**Authors:** Humberto Villacorta Junior, Aline Sterque Villacorta

**Affiliations:** Universidade Federal Fluminense - Pós-graduação em Ciências Cardiovasculares, Niterói, RJ - Brazil

**Keywords:** Hypotension, Blood Pressure, Heart Failure, Renal Insufficiency

## Hypotension, bradycardia and renal dysfunction as obstacles to the treatment of heart failure

"We can easily forgive a child who is afraid of the dark; the real tragedy of life is when men are afraid of light."

*Plato*

Heart failure (HF) is a chronic, high morbidity and high cost disease. The treatment of HF due to left ventricular (LV) systolic dysfunction is well determined and is listed in Medical Guidelines. However, innumerable situations may limit treatment, causing the physician to fail to implement the guidelines. Some serious patients may not tolerate medications or recommended doses; others may have side effects. In some cases, however, there is an excess of caution, failing to prescribe the recommended treatment, fearing complications. The purpose of this article is to demystify, based on the literature, some situations that may prevent the optimized drug treatment from being offered to the HF patient.

The two major side effects that may act as barriers to the treatment of HF are hypotension and worsening renal function. Besides these, we will comment on bradycardia and hyperkalemia.

### Arterial Hypotension

The main limiting factor in the treatment of HF is the lack of understanding of the concept of hypotension in this scenario. Patients with HF due to LV systolic dysfunction, in New York Heart Association (NYHA) functional class III or IV, when adequately medicated, usually have systolic blood pressure (BP) levels as low as 90 mmHg, with no symptoms. In some cases, of non-ischemic etiology, up to 80 mmHg of systolic pressure may be tolerated. A patient of this type, when presented with "normal" BP, 120x80 mmHg, may be submedicated, although these parameters may change, in cases of hypertensive heart disease. Therefore, for the diagnosis of hypotension in these cases, we can not only rely on the absolute value of BP. Symptoms of hypotension, such as lightheadedness, dizziness, weakness, cold hands, asthenia, pre-syncope, or syncope, need to be present.

We must remember that patients with HF have several activated neurohormonal systems, resulting in vasoconstriction (renin-angiotensin-aldosterone system, sympathetic nervous system, endothelin, etc.).^1^ It is therefore necessary that vasodilators be used, to antagonize these effects and reduce afterload, relieving cardiac work. In fact, it is well established in the literature that the use of drugs that combat such systems, such as beta blockers,^2^ inhibitors of conversion enzyme (ACE)^3,4^ or angiotensin receptor blockers (ARBs)^5^ and mineralocorticoid receptor antagonists (spironolactone),^6^ result in increased survival and should be prescribed for all HF patients at the doses recommended in the Medical Guidelines.^7^ Other vasodilators, such as the nitrate-hydralazine combination, have also shown increased survival in a specific setting and may be added to the previous regimen or even replace ACE inhibitor in cases of intolerance or limitations due to renal function.^7,8^

A fall in BP accompanied by symptoms after drug prescription identifies patients of greater severity, since hypovolemia is removed. Nevertheless, an asymptomatic drop in BP with medications used to treat HF may not have a prognostic impact. Indeed, there are data in the literature that suggest that the "lower" BP is actually a marker that treatment is being effective. For example, in the SOLVD study, where enalapril was compared to placebo in patients with HF, systolic BP at study admission averaged 125.3 and 124.5 mm Hg in the enalapril and placebo groups, respectively. At the end of the study, BP fall was greater in the enalapril group than in the placebo group (4.7 vs 4.0 mmHg). However, the survival was higher in the enalapril group, despite a greater fall in PA.^4^ The same was observed in the CONSENSUS study, also with enalapril.^3^ More recently, we highlight the PARADIGM-HF study, where LCZ 696 (valsartan + sacubitril) was compared to enalapril. There was a higher incidence of hypotension in the LCZ 696 group, but the LCZ696 reduced 20% the outcome cardiovascular death and hospitalizations for HF, compared to enalapril.^9^

Therefore, we should not suspend or reduce doses of medications because BP is "low." Only if there are symptoms of hypotension the dose should be reduced. Even in these cases, hypotension is often due to diuretics and not to ACE inhibitors. Check the patient's fluid status. If there are no objective signs of congestion, discontinue the diuretic first, as there may be hypovolemia. Then reduce the dose or stop the nitrate-hydralazine combination. ACE and ARB should be the last ones on the list because their benefits are greater.

### Worsening Renal Function

As observed in the previous section in relation to BP, ACE inhibitors promote increased survival, despite increasing creatinine. In the SOLVD study, the use of enalapril reduced mortality, despite increasing the mean creatinine values^4^ by 0.1 mg/dL. By the mechanism of action of ACEIs, they are expected to increase creatinine, since they promote dilatation of the efferent glomerular artery.^10^ But the final effect is of cardio and renoprotection.^3,4,7,10,11^ There is no definite creatinine value in the literature that contraindicates the use of ACE inhibitors and may even be used in patients on a hemodialysis program,^12^ although they may cause hypotension in this situation. In the SOLVD study, patients were excluded if they had baseline creatinine greater than 2 mg/dL, but the CONSENSUS study included patients with up to 3.4 mg/dL. Increases in creatinine of up to 30% compared to baseline, after the introduction of ACE inhibitors, appear to be safe.^11^ In patients with chronic HF, ACE inhibitors should be prescribed and maintained despite a moderate increase in creatinine provided there is no hyperkalemia or acute renal failure.

In the hospitalized patient with acute HF, it is common to observe transient creatinine elevations during the treatment of congestion with diuretics by reducing intravascular volume. However, as long as congestion has been adequately treated, these increases are not associated with a worse prognosis.^13,14^ In our experience, creatinine, in these cases, usually falls at the end of hospitalization or about 30 days after discharge.^14,15^ In other words, elevated creatinine at admission appears to be a reflection of congestion of the renal veins and transient increases are a consequence of the volume reduction process. Congestion, regardless of worsening of renal function, is associated with worse prognosis.^14,16^ The intense and persistent increase in creatinine seems to indicate a worse prognosis, but transient increases do not.^16^ Therefore, the diuretic and the ACEI should be maintained in this situation, in which there are still evident signs of congestion, despite the increase of slag. Diuretics should only be discontinued in cases of pre-renal renal failure, where creatinine is increased in patients with signs of hypovolemia and ACE inhibitors in cases of severe hyperkalemia or acute renal failure (anuria or oligoanuria associated with increased creatinine).

### Bradycardia

The elevated heart rate (HR) is a marker of severity and is harmful to the patient with HF, and may even be the cause of HF (tachycardiomyopathy).^1,17^ Since the publication of the study Systolic Heart Failure Treatment With the Inhibitor Ivabradine Trial (SHIFT) it is known that HR is not only a marker of severity, but a therapeutic target in HF, since patients treated with ivabradine, an exclusive HR reducer, showed a reduction in the combined outcome of cardiovascular mortality and hospitalization for HF.^18^ Beta blockers prolong the survival of patients with HF and are medicines that reduce HR. Patients with HF should target HR between 50 and 60 bpm. It is not uncommon to find patients with HR above these values, where the maximum dose of the beta-blocker is not achieved, for fear of bradycardia. In another scenario, we see patients with sinus rhythm, already with maximum doses of beta-blockers, with HR above 70 bpm, where ivabradine would be indicated,^7^ but the doctor does not prescribe because of fear of bradycardia. In the US Carvedilol study, there was a higher HR decrease in the carvedilol group compared to placebo (mean, 12.6 vs 1.4 beats, respectively) with a higher incidence of bradycardia (9% vs 1%). However, only 0.9% of patients needed to discontinue carvedilol because of bradycardia. In the SHIFT study, the incidence of asymptomatic and symptomatic bradycardia was, respectively, 6% and 5%. However, the drug was therefore suspended in only 1% of cases. Therefore, we must pursue this target of HR between 50 and 60 bpm. If this target is not reached with beta-blockers, ivabradine may be added if the patient is in sinus rhythm, with systolic dysfunction and HR above 70 bpm. We also recall that digoxin may be an option, used in more severe patients, who remain symptomatic despite treatment with the previous regimen and for frequency control in patients with atrial fibrillation.^7^

### Hyperkalemia

The use of spironolactone in patients with NYHA class III and IV HF resulted in a 30% reduction in the risk of death from any cause.^6^ Subsequently its use was extended to patients in class II, assuming the same benefit found with eplerenone, another aldosterone antagonist, in the EMPHASIS Study.^7^ It is a low-cost medicine with a great impact on HF. Its main side effect is gynecomastia, which occurs in 9% of cases. Another complication that scares the doctor for the potential to cause arrhythmias and sudden death is hyperkalemia. In the RALES study, the incidence of severe hyperkalemia occurred in 10 (1%) patients in the placebo group and 14 (2%) in the spironolactone group, a difference with no statistical significance. However, it is common to hear that in the "real world" the incidence of hyperkalemia would be higher. Many point to a study in Canada that showed increased mortality from hyperkalemia following the publication of the RALES study^19^ to justify their fears. However, a more detailed analysis reveals that often behind the hyperkalemia is inadequate use of spironolactone. For example, a study done in the United States before and after the publication of the RALES study in September 1999 showed that there was a 7-fold increase in the prescription of spironolactone after study publication.^20^ However, in 31% of cases the patient did not meet RALES criteria (had creatinine> 2.5 mg/dL or serum potassium > 5.0 mEq/L). In addition, doses above the recommended dose (25 mg/day) are sometimes used in clinical practice, which increases the risk.

A study in the UK monitored prescriptions for spironolactone between the years 1994 to 2007. There was a marked increase in the prescription of spironolactone following the publication of the RALES Study, but unlike the Canadian study, there was no increase in hospitalizations for hyperkalemia and in outpatients there was a drop in the rates of hyperkalemia, due to the greater monitoring of serum potassium.^21^ These data show the safety of the drug as long as the contraindications are respected and serum potassium and renal function are adequately monitored. The Brazilian guideline for chronic HF does not recommend starting spironolactone if baseline creatinine is above 2.5 mg/dL or serum potassium greater than 5 mEq/L.^7^ Once the drug is started, potassium and creatinine should be monitored frequently. In the RALES study, this was done monthly in the first 3 months and every 3 months in the first year and then every 6 months. We should only suspend spironolactone if there is severe hyperkalemia. The European Society of Cardiology HF Guideline recommends suspension of spironolactone if potassium levels exceed 6.0 mEq/L or if the creatinine exceeds 3.5 mg/dL. For potassium values between 5.6 and 6.0, or creatinine between 2.5 and 3.5 mg/dL, it is recommended to reduce the dose by half and to increase the frequency of monitoring tests.^22^

We hope this article will help to give more confidence to the doctor and increase prescription at the correct doses of medications with benefits in HF. Every medication has a built-in risk of complications, which must be weighed against its benefits. And in the case of HF, these benefits are very well proven.
